# Closing the Loop on Stone Prevention: A Two-Cycle Audit of Recurrence Prevention Counselling After Ureteric Stone Management

**DOI:** 10.7759/cureus.116057

**Published:** 2026-09-10

**Authors:** Muhammad Danial Bin Rosli, Nadeem Iqbal

**Affiliations:** 1 Department of Urology, Altnagelvin Area Hospital, Londonderry, GBR

**Keywords:** clinical audit, guideline adherence, medical expulsive therapy, patient information leaflet, quality improvement, secondary prevention, ureteric stones, urolithiasis

## Abstract

Background

National guidance requires that every patient presenting with a new or recurrent urinary stone receives documented dietary and fluid advice, and this is a measurable quality indicator rather than a discretionary extra. In our department, this advice appeared to be delivered inconsistently, particularly at acute presentation, but the reasons were unclear. We audited compliance against national standards and, unusually for an audit of this kind, surveyed the clinicians responsible for delivering the advice to establish why the standard was being missed before attempting to correct it.

Methodology

We conducted a two-cycle retrospective audit in a district general hospital urology department. Cycle one reviewed 59 consecutive patients presenting with ureteric stones between February and April 2026. Compliance was assessed against national guideline standards for emergency computed tomography within 24 hours, alpha-blocker therapy in distal ureteric stones smaller than 10 mm, metabolic evaluation, and documented dietary and fluid advice. An anonymous survey of eight clinicians examined guideline awareness, self-reported practice, documentation habits, perceived barriers, and confidence. Interventions followed: a standardised printed prevention leaflet across the emergency department and wards, an explicit prompt within discharge documentation, written clarification of who delivers advice at each point in the pathway, and departmental teaching. Cycle two re-audited 46 consecutive patients between June and July 2026 using an identical proforma.

Results

Cycle one revealed near-total failure of the prevention standard. Documented prevention advice and dietary advice were each present in 22.0% of patients, and hydration advice in 18.6%. Metabolic evaluation was arranged in 35.6%, and alpha-blocker therapy was prescribed to only five of 14 patients meeting the guideline indication. Imaging and follow-up performed well at 96.7% and 89.8%, respectively, indicating a deficit specific to prevention rather than a general failure of care. The survey proved decisive: every respondent reported rarely documenting advice, 87.5% identified unclear professional responsibility as the principal barrier, and mean self-rated confidence was 2.0 of 5. Cycle two showed improvement across every prevention measure. Documented prevention advice and dietary advice each rose to 89.1%, hydration advice to 100%, and metabolic evaluation to 89.1%. Alpha-blocker therapy was prescribed to 15 of 17 eligible patients, rising to 88.2%. Imaging reached 100% and follow-up 95.7%. The gap between acute and elective settings closed, with documented advice rising from 20.0% to 88.0% in emergency presentations and from 24.1% to 90.0% in elective patients.

Conclusions

Poor compliance here reflected system design rather than clinical knowledge or intent. Surveying staff before intervening identified diffuse responsibility and absent written resources as the operative barriers, and compliance subsequently improved by 53 to 81 percentage points across every prevention measure. We cannot demonstrate that the intervention package caused these changes, and the design does not permit attribution to any individual component. Audits reporting non-compliance without investigating its cause are of limited value; a brief staff survey is a proportionate, high-yield addition to the audit cycle.

## Introduction

Few surgical conditions punish the patient twice as reliably as urinary stone disease. The first insult is the colic that brings them to the emergency department; the second arrives quietly, months or years later, because nothing done to remove that stone made the next one any less likely. Recurrence has been reported at up to 50% by five years and 80% to 90% by 10 years, and surgical clearance confers no protection against the underlying lithogenic tendency [1]. Each recurrence carries its own admission, its own imaging, its own theatre episode, and its own risk to renal function [1]. The stone in front of us is a symptom. The disease is metabolic, dietary, and behavioural, and it outlives the operation by decades.

This is not a controversial position, nor a recent one. Nearly 30 years ago Borghi and colleagues randomised patients with idiopathic calcium nephrolithiasis to a simple high fluid intake target and followed them for five years, halving recurrence from 27% to 12.1% and lengthening the interval to recurrence in those who complied [2]. No drug, no device, and no operative refinement in the intervening decades has produced a preventive effect that cheap or that reproducible. Later systematic review of randomised trials confirmed fluid intake as the most secure of the dietary interventions, with sodium restriction, moderation of animal protein, and selected pharmacological therapy occupying supporting roles [3]. The preventive evidence base is, by surgical standards, mature.

United Kingdom guidance reflects this without ambiguity. National Institute for Health and Care Excellence guideline NG118 states at section 1.8.1 that adults, children, and young people with renal or ureteric stones should be given information on diet and fluid intake to prevent recurrence, in a format they can actually understand [4]. Section 1.7 governs metabolic assessment, and section 1.3.1 recommends considering an alpha blocker for distal ureteric stones smaller than 10 mm [4]. Quality standard QS195 then does something more uncomfortable: it converts prevention advice from good intention into a measurable indicator, with an expectation that clinicians are trained to give it and that written material exists to support them [5]. The denominator is every patient with a stone. There is no cohort in whom the standard does not apply.

The literature suggests we are collectively poor at meeting it. Adherence to preventive measures among stone formers sits at roughly 30%, with inadequate patient education repeatedly implicated [6]. Clinician performance is arguably worse: in a series of high-risk stone formers undergoing surgery, only 3.8% satisfied every guideline metric for metabolic evaluation and prevention, with losses accumulating at each step of the pathway [7]. United Kingdom departments have published comparable baseline failures for biochemical testing [8] and for the radiological investigation of renal colic [9], and in both cases targeted intervention produced substantial improvement. Poor compliance with stone guidance is evidently correctable. The difficulty lies in knowing what to correct.

Here conventional audit tends to disappoint. It is very good at establishing that something was not done and almost silent on why, which leaves the improvement phase to guesswork. A department confronted with a low prevention advice rate will often reach for teaching, when the operative barrier may be that no leaflet exists, or that every clinician in the pathway assumes the task belongs to somebody further downstream. We therefore built the diagnosis of cause into the audit itself. Alongside a retrospective review of compliance with NG118 in patients presenting with ureteric stones, we surveyed the clinicians responsible for delivering that care about their guideline awareness, actual practice, documentation habits, perceived barriers, and confidence. The question was whether an intervention package assembled from what clinicians told us, rather than from what we assumed, could shift compliance against national standards within a single cycle.

## Materials and methods

This study was conducted as a two-cycle retrospective clinical audit within the urology department at Altnagelvin Area Hospital, a district general hospital in Northern Ireland serving both an unselected emergency take and an elective stone service. The design was deliberately closed-loop, and deliberately unconventional in one respect. Rather than measure compliance, assume a cause and intervene, we built the diagnosis of cause into the first cycle itself by surveying the clinicians responsible for delivering the care at the same time as we audited their records. Reporting follows the principles set out in the Standards for Quality Improvement Reporting Excellence (SQUIRE) [10].

The project was registered with the trust clinical audit department before data collection. Formal ethical approval was waived on the basis that the work constituted service evaluation against established national standards, involved no alteration to patient care, and used no patient-identifiable data beyond that already held within the clinical record.

The first cycle comprised 59 consecutive patients presenting with ureteric stones between February and April 2026. Cases were identified from urology theatre lists, emergency admission records, and outpatient clinic records, a triangulated approach chosen because stone patients enter the service by several routes and any single source would have systematically under-captured one of them. Adults aged 18 years or over with a radiologically confirmed ureteric or renal stone during the study window were eligible. Patients under 18 years and pregnant patients were not eligible, the latter because the imaging and management pathway in pregnancy diverges materially from that assessed by the standards under audit; in practice, neither group presented during either window, and no patient was excluded on any ground. All identified cases were therefore analysed, and the denominators reported here represent complete consecutive series.

Data were extracted from case notes and electronic records against a purpose-built proforma. Recorded variables comprised age, sex, presentation setting, stone site and maximum diameter, imaging modality, presence of hydronephrosis, analgesia administered, medical expulsive therapy and agent used, definitive management, admission status, documented hydration advice, documented dietary advice, arrangement of metabolic evaluation, follow-up arrangements, and any previous history of stone disease. A free-text field captured where and how advice had been delivered. This last field earned its place: it revealed that advice, when given at all, clustered in the elective clinic rather than at the acute presentation, which shaped the interventions that followed.

Audit standards were drawn from National Institute for Health and Care Excellence guideline NG118 and quality standard QS195, with European Association of Urology guidance used as a secondary reference where NG118 is less prescriptive on dietary specifics [1,4,5]. Four standards carried the analysis. Section 1.1.1 requires computed tomography of the kidneys, ureters, and bladder within 24 hours of emergency presentation. Section 1.3.1 recommends considering an alpha blocker for distal ureteric stones smaller than 10 mm; eligibility was defined as a stone in the distal ureter measuring under 10 mm on index imaging. Section 1.7 governs metabolic evaluation. Section 1.8.1 requires documented dietary and fluid advice for every patient, with no exempt subgroup. A local target of 90% was applied to each standard for reporting purposes.

One methodological decision deserves stating plainly, because it determines how the headline figures should be read. Compliance was scored strictly on what appeared in the clinical record. Advice delivered verbally but not documented was counted as non-compliant. This is not pedantry: QS195 explicitly frames the provision of information as something that must be evidenced, and undocumented advice is invisible to the next clinician in the pathway, unavailable to the patient on discharge, and indistinguishable from advice never given [5]. Some proportion of our first-cycle failures will represent recording rather than clinical omission, and we address this directly in the discussion.

Running alongside the first cycle, an anonymous electronic survey was distributed to all medical staff working within the department. The instrument was developed locally, as no validated tool exists for this question, and comprised items on grade, the clinical settings in which stone patients were encountered, familiarity with NG118, the timing and content of self-reported prevention advice, frequency of documentation, practice regarding metabolic evaluation in recurrent stone formers, perceived barriers as a multiple-response item, self-rated confidence on a five-point Likert scale, and two free-text items inviting suggestions for improvement. Participation was voluntary, and no identifying data were collected. Eight responses were received, spanning foundation, core and senior trainee, or specialty doctor grades. The full instrument is reproduced in the Appendices.

The intervention package was assembled only after both the first-cycle results and the survey findings had been presented to the department in mid-May 2026, and each component maps onto a barrier that clinicians themselves had nominated rather than one we had inferred. A standardised printed stone prevention leaflet was introduced and stocked across the emergency department and urology wards, addressing the request that dominated the free-text responses. Prevention advice was added as an explicit item within discharge and post-procedure documentation. Written clarification was circulated defining which clinician delivers prevention advice at each point in the pathway: emergency assessment, ward round, preoperative review, postoperative discharge, and outpatient clinic, targeting the barrier most frequently cited. A departmental teaching session covered the NG118 requirements for metabolic evaluation, prevention advice, and medical expulsive therapy. This sequencing matters to the interpretation of our results: the interventions were not selected from a menu of plausible quality improvement measures but derived from stated obstacles, and the same survey that identified them provides the outcome logic against which their effect can be judged [11]. All intervention components were implemented over the following two weeks.

The second cycle reviewed 46 consecutive patients presenting between June and July 2026, applying the same proforma, standards, case ascertainment method, and eligibility criteria as the first. Data from both cycles were compiled in Microsoft Excel (Microsoft Corp., Redmond, WA, USA). Compliance for each standard is reported as a proportion of the eligible denominator with absolute numerators given, and cycle-to-cycle change is expressed in percentage points. Given the sample sizes and the descriptive intent of a clinical audit, no inferential statistical testing was undertaken, and no power calculation was performed; readers should treat the differences reported as observed change in a service evaluation rather than as effects estimated with confidence intervals.

## Results

A total of 59 patients were audited in the first cycle and 46 in the second, with no exclusions in either. Baseline characteristics were closely comparable between cohorts in age, sex distribution, stone size, and the balance of emergency to elective presentation (Table 1). Two differences deserve note. Stone distribution shifted, with renal stones accounting for 10 patients in the first cycle and none in the second, where a single bladder stone appeared instead. More consequentially, the proportion with recurrent stone disease fell from 18 (30.5%) patients to two (4.3%) patients, which constrains subgroup interpretation and is addressed below.

**Table 1:** Baseline characteristics of both audit cohorts. Percentages are of the total cohort for each cycle. No patients were excluded from either cycle. The abbreviation n denotes the number of patients, and mm denotes millimetres.

Characteristic	Cycle 1 (n = 59)	Cycle 2 (n = 46)
Median age, years (range)	62 (21–85)	58 (25–94)
Male, n (%)	32 (54.2)	26 (56.5)
Female, n (%)	27 (45.8)	20 (43.5)
Median stone diameter, mm (range)	7.25 (3.5–20.0)	8.0 (3.0–20.0)
Emergency presentation, n (%)	30 (50.8)	25 (54.3)
Elective presentation, n (%)	29 (49.2)	20 (43.5)
Urology clinic presentation, n (%)	0 (0.0)	1 (2.2)
Upper ureteric stone, n (%)	30 (50.8)	20 (43.5)
Mid ureteric stone, n (%)	3 (5.1)	6 (13.0)
Distal ureteric stone, n (%)	16 (27.1)	19 (41.3)
Renal stone, n (%)	10 (16.9)	0 (0.0)
Bladder stone, n (%)	0 (0.0)	1 (2.2)
Hydronephrosis on index imaging, n (%)	41 (69.5)	38 (82.6)
Admitted, n (%)	47 (79.7)	37 (80.4)
Recurrent stone disease, n (%)	18 (30.5)	2 (4.3)

Imaging in the first cycle comprised computed tomography of the kidneys, ureters, and bladder in 49 patients; computed tomography of the abdomen and pelvis in seven; and mercaptoacetyltriglycine renography in one. All 46 patients in the second cycle underwent computed tomography of the kidneys, ureters, and bladder. Definitive management in the first cycle comprised ureteric stent insertion alone in 26 patients, ureteroscopy with laser fragmentation with or without stent placement in 30, one of which required multiple sessions, stent insertion with laser in two, and stone basket retrieval in one. In the second cycle, this comprised ureteroscopy with laser fragmentation in 21 patients, stent insertion in 11, shockwave lithotripsy in three, stone basket retrieval in one, nephrostomy in one, and conservative management in two.

The first cycle exposed a service performing two quite different jobs to two quite different standards (Table 2, Figure 1). Diagnostic and organisational measures were sound. Computed tomography of the kidneys, ureters, and bladder was performed in 29 of 30 (96.7%) emergency presentations, and follow-up was arranged for 53 of 59 (89.8%) patients, comprising a further procedure or operative session in 32 patients, clinic review in 13, and interval imaging in eight. Preventive measures were not sound. Documented prevention advice was present in 13 of 59 (22.0%) records, dietary advice in 13 (22.0%), hydration advice in 11 (18.6%), and metabolic evaluation was arranged in 21 (35.6%) patients. Analgesia was documented in 39 of 59 (66.1%) patients.

**Table 2:** Compliance with national guideline standards across both audit cycles. Eligibility for alpha-blocker therapy was defined as a distal ureteric stone measuring under 10 mm, and the denominator for that row reflects eligible patients only. The denominator for computed tomography reflects emergency presentations only. The abbreviation n denotes the number meeting the standard, N the eligible denominator, pp percentage points, and CT KUB computed tomography of the kidneys, ureters, and bladder.

Standard	Cycle 1 n/N (%)	Cycle 2 n/N (%)	Change (pp)
Documented prevention advice	13/59 (22.0)	41/46 (89.1)	+67.1
Hydration advice given	11/59 (18.6)	46/46 (100.0)	+81.4
Dietary advice given	13/59 (22.0)	41/46 (89.1)	+67.1
Metabolic evaluation arranged	21/59 (35.6)	41/46 (89.1)	+53.5
Alpha-blocker therapy, eligible patients	5/14 (35.7)	15/17 (88.2)	+52.5
Alpha blocker therapy, whole cohort	27/59 (45.8)	34/46 (73.9)	+28.1
Analgesia documented	39/59 (66.1)	42/46 (91.3)	+25.2
CT KUB in emergency presentations	29/30 (96.7)	25/25 (100.0)	+3.3
Follow-up arranged	53/59 (89.8)	44/46 (95.7)	+5.9

**Figure 1:**
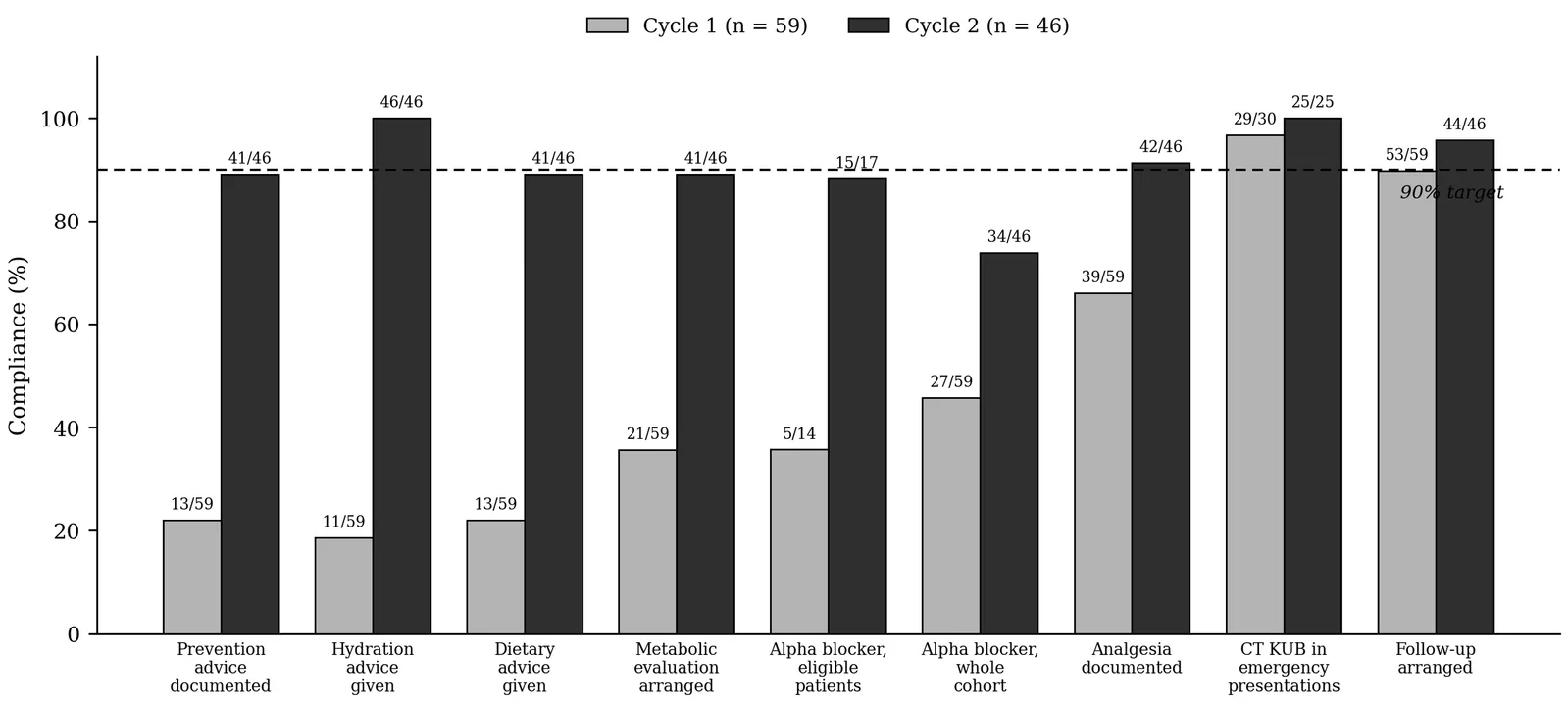
Compliance with national guideline standards across both audit cycles. Numerators and denominators are shown above each bar. The dashed horizontal line indicates the locally agreed 90% compliance target. Alpha-blocker therapy is presented twice, once restricted to patients meeting the guideline indication of a distal ureteric stone under 10 mm and once across the whole cohort. The abbreviation CT KUB denotes computed tomography of the kidneys, ureters, and bladder, and n denotes the number of patients in each cycle.

Alpha-blocker therapy requires two denominators to interpret honestly. Across the whole first-cycle cohort, 27 (45.8%) patients received it. Among the 14 patients meeting the guideline indication of a distal ureteric stone measuring under 10 mm, only five (35.7%) did. Every prescription in both cycles was tamsulosin, so the deficit lay in whether the drug was given at all rather than in agent selection. That the same department imaged 96.7% of its emergency presentations correctly while documenting prevention advice for 22.0% argues against a general failure of care and in favour of a specific, structural failure confined to prevention.

The subgroup findings sharpened this. In total, 18 patients in the first cycle had documented previous stone disease, the group for whom prevention guidance is most emphatic, yet only six (33.3%) received documented prevention advice and only 10 (55.6%) had metabolic evaluation arranged. Where advice was recorded at all, it clustered downstream: seven of the 13 documented instances occurred in elective patients, and the free-text field located most of these in follow-up clinic rather than at the index presentation. Only six of 30 (20.0%) emergency presentations carried any documented advice, against seven of 29 (24.1%) elective presentations (Figure 2). The acute contact, which is the one moment every stone patient reliably attends, was the point at which prevention was least likely to happen.

**Figure 2:**
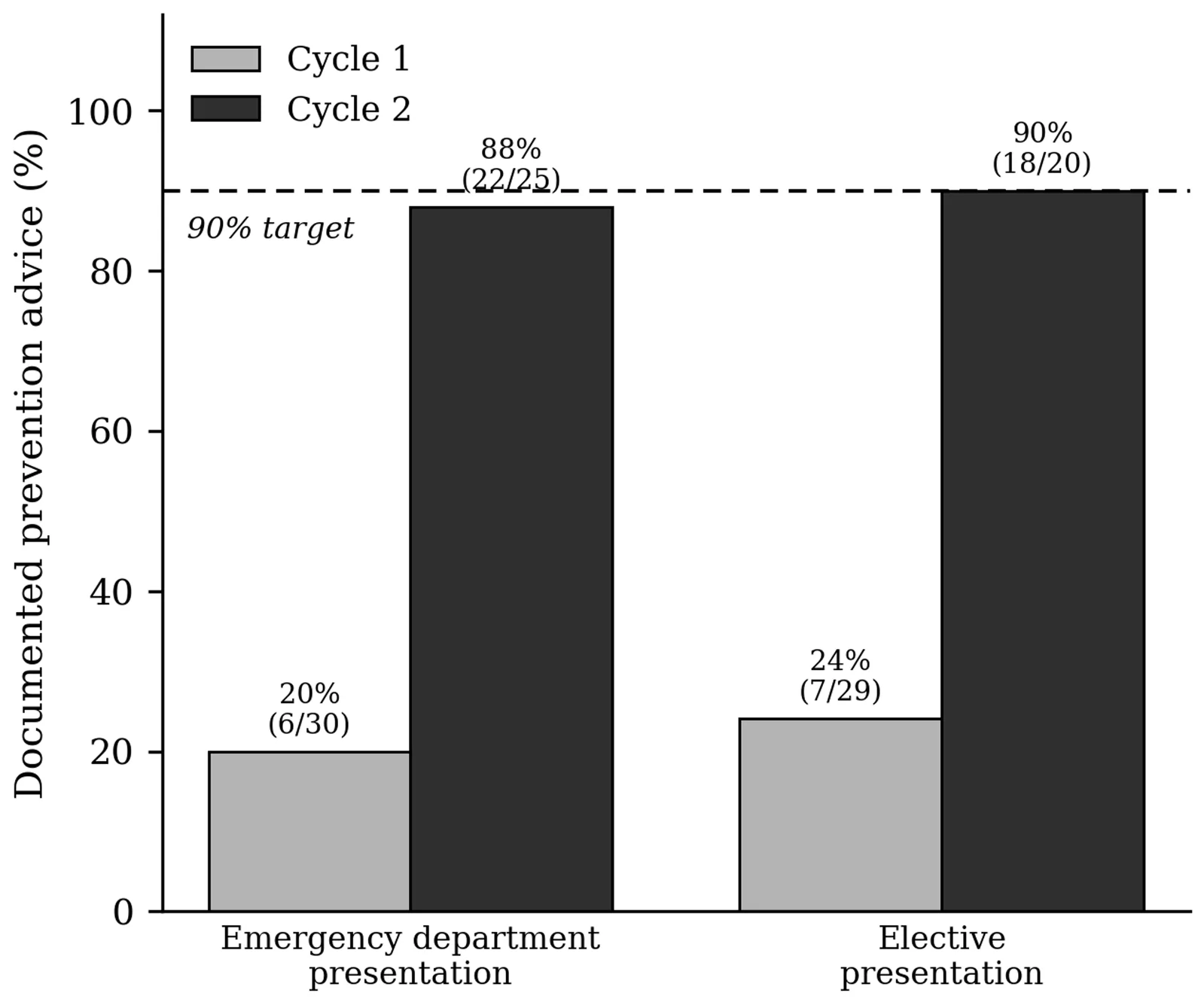
Documented prevention advice by presentation setting. In cycle 1, prevention advice was documented for six of 30 emergency presentations and seven of 29 elective presentations. In cycle 2, this rose to 22 of 25 and 18 of 20, respectively, closing the gap between the acute and elective pathways. One patient in cycle 2 presented through the urology clinic and is not shown. The dashed horizontal line indicates the locally agreed 90% compliance target.

The clinician survey, returned by eight respondents spanning foundation, core, and senior grades, explained why. The full instrument and complete response data are provided in tabular form in the Appendices. The single most striking finding was unanimous: all eight respondents reported that they rarely document prevention advice, with none selecting “always” or “sometimes.” Guideline familiarity was limited, with five (62.5%) respondents aware of the guideline but not having read it in detail and one (12.5%) unaware of it entirely. Asked when they give prevention advice, three (37.5%) selected the ward round and two (25.0%) the outpatient clinic, while three (37.5%) conceded they do not routinely give it at all. Not one respondent reported giving advice at emergency assessment or clerking, precisely mirroring the documentation pattern observed in that setting. Content was patchy: fluid intake was covered by six (75.0%) respondents, but sodium restriction and animal protein moderation were covered by none.

On barriers, the responses were unusually decisive (Figure 3). Unclear professional responsibility was cited by seven of eight (87.5%) respondents, ahead of competing clinical priorities (five, 62.5%), absence of a standard leaflet (four, 50.0%) and uncertainty about what advice to give (four, 50.0%). Mean self-rated confidence in delivering prevention advice was 2.0 on a five-point scale, with six (75.0%) respondents scoring themselves at 2 and none scoring above 3 (Figure 4). All five free-text responses, without prompting, requested either a printed leaflet or clarity about whose job the task was. Metabolic evaluation practice in recurrent stone formers showed the same diffusion of responsibility: four (50.0%) respondents reported rarely arranging it and leaving it to follow-up clinic, and one (12.5%) was unsure of the pathway altogether.

**Figure 3:**
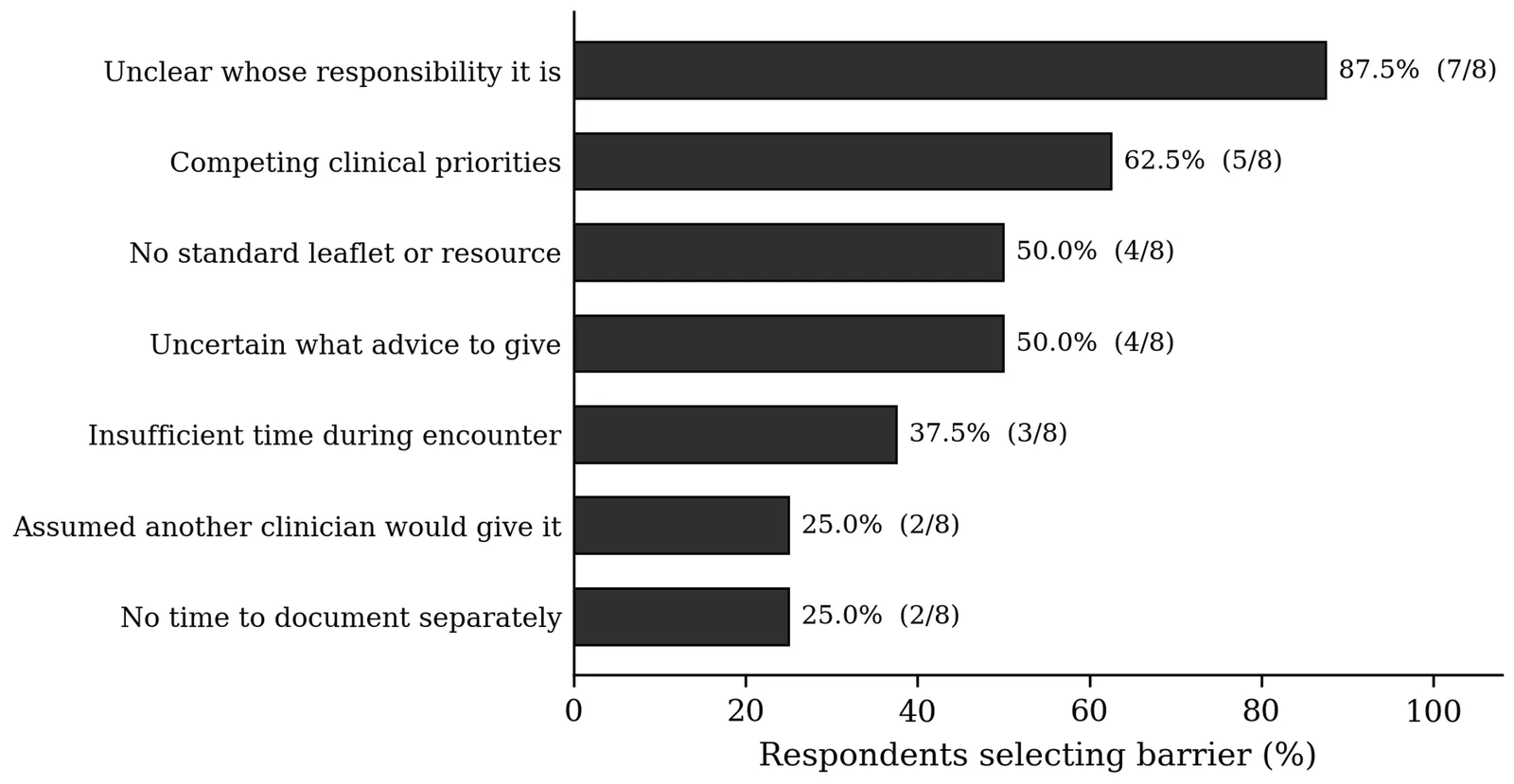
Clinician-reported barriers to delivering secondary prevention advice. Responses from eight departmental clinicians surveyed during cycle 1. The item permitted multiple responses, so percentages sum to more than 100%. Unclear professional responsibility was the most frequently selected barrier, cited by seven of eight respondents. No respondent reported experiencing no significant barriers. The abbreviation n denotes number of respondents.

**Figure 4:**
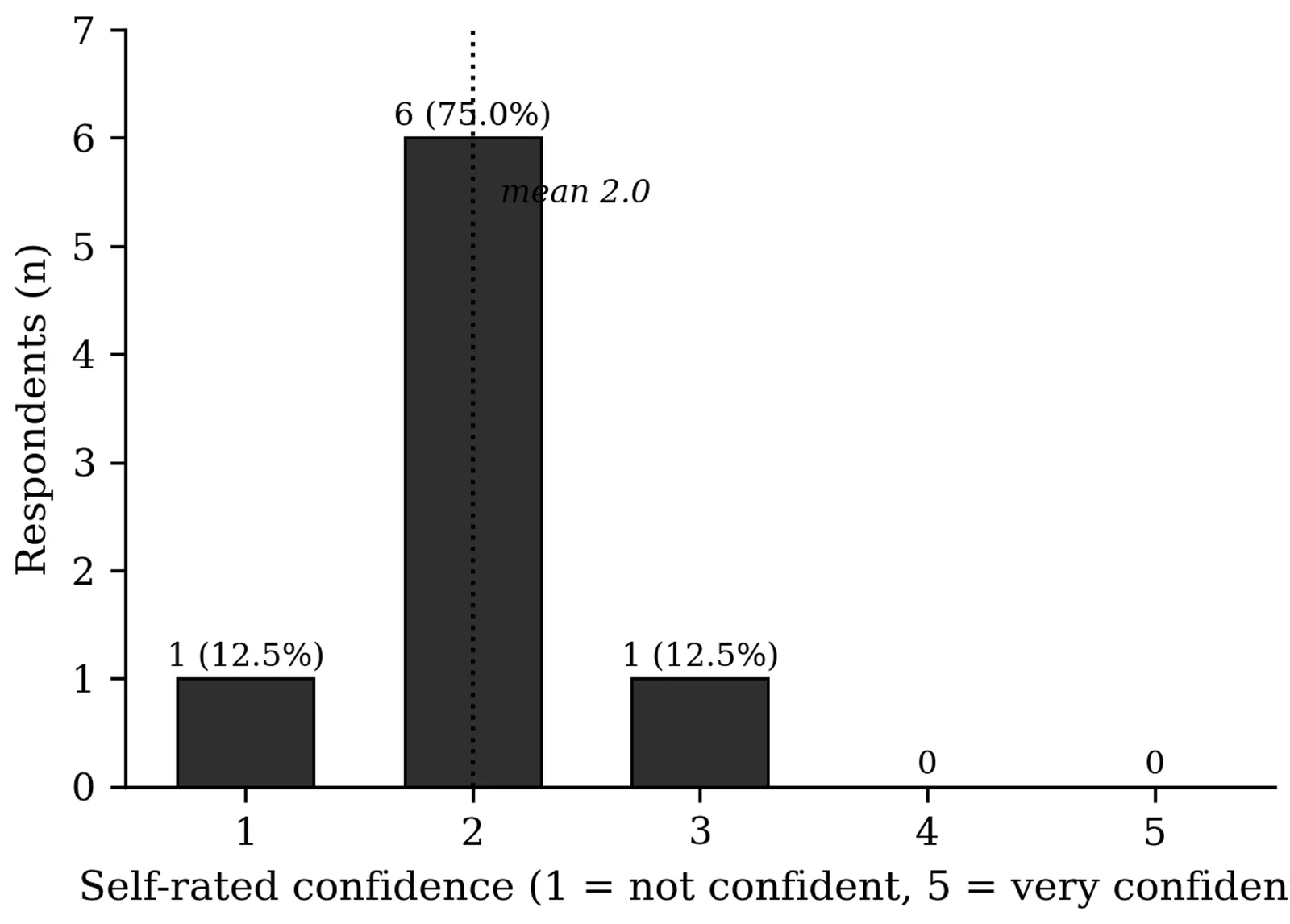
Distribution of clinician self-rated confidence in delivering stone prevention advice. Respondents rated confidence on a five-point scale where 1 indicated not confident and 5 indicated very confident. Six of eight respondents scored themselves at 2 and none scored above 3, giving a mean of 2.0. The dotted vertical line marks the mean. The abbreviation n denotes number of respondents.

The second cycle showed improvement across every standard (Table 2, Figure 1). Documented prevention advice and dietary advice each rose from 22.0% to 89.1%, an increase of 67.1 percentage points. Hydration advice reached all 46 (100.0%) patients, rising by 81.4 percentage points. Metabolic evaluation also reached 41 (89.1%) patients, a gain of 53.5 percentage points. Alpha-blocker therapy rose to 34 (73.9%) patients across the whole cohort and to 15 of 17 (88.2%) guideline-eligible patients. Analgesia documentation improved from 66.1% to 91.3%. The two measures already performing well improved further, with computed tomography reaching all 25 (100.0%) emergency presentations and follow-up in 44 of 46 (95.7%) patients, comprising a further procedure in 26 patients, interval imaging in 12, clinic review in one, and one patient who died before follow-up.

The distributional change mattered as much as the aggregate. Documented advice in emergency presentations rose from six of 30 (20.0%) to 22 of 25 (88.0%), and in elective patients, from seven of 29 (24.1%) to 18 of 20 (90.0%) (Figure 2). The gap between the acute and elective pathways, which was the defining feature of the first cycle, effectively closed, and both settings reached or approached the 90% target.

A limitation of the second cycle should be stated alongside its results. Only two patients had a history of recurrent stone disease, compared with 18 in the first cycle (Table 1). Both received prevention advice, and both had metabolic evaluation arranged, but a denominator of two supports no conclusion about the recurrent stone subgroup, and the improvement in metabolic evaluation across the whole cohort cannot therefore be attributed specifically to better care of recurrent stone formers.

## Discussion

Compliance with the prevention standard in this department improved substantially across every measure between the two cycles, and without additional staffing or funding. The temptation is to attribute that movement to the interventions and stop there. Our design does not permit it. This was an uncontrolled before-and-after comparison in which four interventions were introduced together, and secular trend, the effect of the audit process itself, and the contribution of any individual component cannot be separated from one another. What follows is therefore an account of what changed and what we think is the most plausible reading, not a claim of demonstrated effect.

Our tentative answer is that the survey shaped what we chose to do. Conventional audit tells a department that it is non-compliant and leaves it to infer why, and departments overwhelmingly infer a knowledge deficit because education is the intervention closest to hand. Had we followed that instinct, we would have taught, and we suspect we would have achieved less, because teaching was not what our clinicians said they needed. Seven of eight respondents identified unclear professional responsibility as their principal barrier, and half cited the absence of any leaflet to hand over. Only one in eight lacked awareness of NG118 altogether. The obstacle was not that clinicians did not know prevention mattered; it was that no one believed it was specifically their job, and no one had anything to give the patient. Ivers and colleagues, in the Cochrane review of audit and feedback, found effects that were generally small and highly variable, with impact depending substantially on how feedback was delivered and what accompanied it [11]. Our experience is at least consistent with the possibility that audits which diagnose their own failure mode fare better than those that do not, though a single uncontrolled cycle cannot establish this.

The pattern of the first-cycle failure supports the diagnosis. Prevention advice was not uniformly absent; it was absent at the acute presentation and present, patchily, in elective clinic. Only six of 30 emergency patients had documented advice against seven of 29 elective patients, and no survey respondent reported advising on emergency assessment or clerking. This is the signature of diffused responsibility rather than ignorance: each clinician in the pathway appeared to assume the task belonged to someone downstream. That the same department imaged 96.7% of its emergency presentations correctly in the same cycle makes a general standards failure implausible.

Our baseline figures sit comfortably within the published range, which is itself a sobering observation. Hunt and colleagues found that only 3.8% of high-risk stone formers met every guideline metric for metabolic evaluation and prevention, with attrition at each step [7]. Deligiannis and colleagues report patient adherence to dietary prevention at approximately 30%, noting inadequate education as a contributor [6]. United Kingdom departments have published comparable baseline deficits and comparable post-intervention gains for biochemical testing [8] and for the imaging of renal colic [9]. Prevention appears to be the reliably neglected limb of stone care across health systems, notwithstanding that the evidence supporting it is older and arguably more secure than the evidence for much of what we do operatively. Borghi et al.’s trial halved recurrence with water [2], and Fink et al.’s synthesis confirmed fluid intake as the most robust of the dietary interventions [3]. We are not failing to deliver a marginal intervention.

The design does not permit attribution of effect to individual components. Four interventions were introduced simultaneously, and the changes in alpha-blocker prescribing and metabolic evaluation are as plausibly related to the teaching session as the change in hydration advice is to the leaflet. There is a temporal logic to the mapping we propose, in that hydration advice reaching 100% followed introduction of a leaflet whose first section addresses fluid intake, but this remains an inference. A stepped introduction would have permitted attribution at the cost of delaying benefit, and in a service evaluation we judged the trade unfavourable.

Two threats to the durability of these findings deserve naming. The first is the Hawthorne effect: clinicians knew an audit was running, and awareness of observation can itself alter behaviour, though the systematic review evidence suggests the magnitude is heterogeneous and difficult to quantify rather than uniformly large [12]. The second is more prosaic and, in our view, more dangerous. Every component of our intervention package depends on continuity: leaflets must be restocked, documentation prompts must survive template revisions, and the clarification of responsibility must be transmitted to clinicians who rotate every four to six months. Improvement achieved through local memory decays when the people holding that memory leave. This is why our recommendations centre on embedding the requirements in departmental induction rather than relying on the teaching session that produced the immediate gain.

Further limitations should be weighed. The second cycle covered a shorter window than the first, giving a smaller cohort, and included only two patients with recurrent stone disease against 18 in the first cycle. The improvement in metabolic evaluation across the whole cohort therefore cannot be read as improved care of recurrent stone formers specifically, and sections 1.8.3 to 1.8.5 of NG118, which govern potassium citrate and thiazide therapy in that group, remain effectively unaudited. Data were collected retrospectively by a single investigator without an inter-rater reliability check. Compliance was assessed on documentation alone, so advice genuinely delivered but unrecorded was scored as failure, defensible against the QS195 requirement that provision be evidenced [5], but it means our first-cycle figures describe recorded practice rather than actual practice, and the true baseline was probably somewhat better than 22%. Finally, this is a single department in one hospital, and eight survey respondents is a small denominator on which to build any account of causation, however unanimous their responses.

What we would offer other units is not the specific intervention package, which is unremarkable and locally contingent, but the sequence. Audit the records and survey the clinicians in the same cycle, then build the intervention from what the clinicians say rather than from what the numbers imply. The survey took minutes to complete, cost nothing, and identified an obstacle, unclear ownership, that no amount of records review would have revealed. Reporting here follows SQUIRE 2.0 [10], and we would suggest that its emphasis on describing the reasoning behind an intervention is easier to satisfy when that reasoning is derived from data rather than assumption.

## Conclusions

Secondary prevention is the part of stone care most easily deferred and most consistently neglected, and this audit found a department documenting prevention advice for barely one patient in five while imaging almost every emergency presentation correctly. That contrast matters more than either figure alone, because it locates the failure precisely. This was not a unit performing poorly across the board; it was a unit in which one specific standard had no owner. The clinician survey made that explicit, with seven of eight respondents identifying unclear professional responsibility as their principal obstacle and half reporting that no patient information existed to hand over. Had we assumed a knowledge deficit and responded with teaching alone, we would have addressed a problem our clinicians did not have. Following an intervention package built from stated rather than inferred barriers, compliance improved across every prevention standard, with documented advice rising from just over a fifth of patients to three-quarters, hydration advice reaching every patient audited, and alpha-blocker therapy for eligible distal stones rising from a third to nearly nine in ten. We cannot demonstrate that the package caused these changes, and we do not claim it. A residual inconsistency persists in our summary documentation field, which we interpret as a recording artefact and have addressed by simplifying that field to a single confirmatory entry. The durability of these gains is not assured: leaflets require restocking, documentation prompts require protection through template revisions, and clinicians rotate out of the department every few months carrying the local knowledge with them, which is why embedding these requirements in departmental induction matters more than the teaching session that produced the immediate effect. For units contemplating similar work, our proposal is methodological rather than clinical. Survey the clinicians in the same cycle as the records, and let what they tell you inform what you change. It costs almost nothing, and it may be the difference between an audit that documents failure and one that acts on the right cause.

## Appendices

Appendix 1. Clinician survey instrument

The questionnaire below was developed locally for this audit, as no validated instrument exists to assess clinician-level barriers to delivering secondary prevention advice in urolithiasis. It was distributed electronically to all medical staff in the urology department during cycle one. Participation was voluntary and anonymous, and no identifying data were collected. Eight responses were received.

**Table 3:** Clinician survey instrument as distributed during cycle one. Items are grouped by section: role and setting (1), guideline awareness (2), delivery of prevention advice (3), recurrent stone formers (4), barriers and confidence (5), and free-text items (6). Single denotes one permitted response, and Multiple denotes that more than one option could be selected.

Item	Question	Response options	Response type
1.1	What is your current grade?	Foundation Year 1 Foundation Year 2 or Core Trainee Specialty Registrar or Specialty and Associate Specialist doctor Consultant Nursing staff	Single
1.2	In which clinical settings do you regularly see patients with ureteric stones?	Emergency department or on-call Urology ward round Admissions clerking Outpatient or elective clinic	Multiple
2.1	How familiar are you with the national guideline on renal and ureteric stones?	Not aware of it Aware but not read in detail Familiar with key recommendations Use it regularly in practice	Single
2.2	Were you aware that the guideline requires all patients with a new or recurrent stone to receive documented dietary and fluid advice?	Yes No Unsure	Single
2.3	Were you aware that the guideline recommends considering an alpha blocker for distal ureteric stones smaller than 10 millimetres?	Yes No Unsure	Single
3.1	When you give prevention advice to a patient with a ureteric stone, at what point does this most commonly happen?	During emergency department assessment or admission clerking, verbally During ward round Post-operatively, prior to discharge In outpatient or follow-up clinic I do not routinely give this advice	Multiple
3.2	Which of the following prevention topics do you typically cover?	Fluid intake Dietary salt restriction Calcium and dairy intake advice Low-oxalate dietary advice Animal protein moderation Weight management or physical activity Increased citrus or lemon juice intake I do not routinely cover prevention topics	Multiple
3.3	How often is this documented in the ward round entry, discharge letter, post-operative plan or clinic letter?	Always Sometimes, if I remember Rarely Never	Single
4.1	For a patient presenting with their second or subsequent stone, do you routinely arrange or refer for metabolic evaluation?	Yes, always Sometimes, if I remember Rarely — left to follow-up clinic No, I am not sure of the pathway	Single
5.1	What are the main barriers to giving prevention advice to patients?	Not enough time during the encounter No standard leaflet or resource available Unsure of what advice to give Unclear whose responsibility it is Other clinical priorities take precedence Patient in too much pain or distress to engage Assumed it would be given by someone else No time to document it separately I do not experience significant barriers	Multiple
5.2	On a scale of 1 to 5, how confident do you feel giving ureteric stone prevention advice to patients?	1 (not confident) to 5 (very confident)	Five-point scale
6.1	What single change would most improve the delivery of stone prevention advice in your department?	Open response	Free text
6.2	Any other comments?	Open response	Free text

Appendix 2. Survey responses in full

Percentages are of eight respondents throughout. Multiple-response items sum to more than 100%.

**Table 4:** Respondent characteristics, guideline awareness, and reported practice. Responses from eight departmental clinicians surveyed during cycle one. The abbreviation n denotes the number of respondents selecting that option.

Item	Response	N	%
Grade	Foundation Year 2 or Core Trainee	5	62.5
	Specialty Registrar or Specialty and Associate Specialist doctor	2	25.0
	Foundation Year 1	1	12.5
Clinical setting	Emergency department or on-call	7	87.5
	Urology ward round	7	87.5
	Admissions clerking	6	75.0
	Outpatient or elective clinic	4	50.0
Guideline familiarity	Aware but not read in detail	5	62.5
	Familiar with key recommendations	2	25.0
	Not aware of it	1	12.5
	Use it regularly in practice	0	0.0
Timing of advice	During ward round	3	37.5
	Do not routinely give this advice	3	37.5
	In outpatient or follow-up clinic	2	25.0
	Post-operatively, prior to discharge	1	12.5
	At emergency assessment or clerking	0	0.0
Topics covered	Fluid intake	6	75.0
	Do not routinely cover prevention topics	4	50.0
	Calcium and dairy intake advice	3	37.5
	Weight management or physical activity	3	37.5
	Low-oxalate dietary advice	1	12.5
	Increased citrus or lemon juice intake	1	12.5
	Dietary salt restriction	0	0.0
	Animal protein moderation	0	0.0
Documentation frequency	Rarely	8	100.0
	Always, Sometimes or Never	0	0.0
Metabolic evaluation in recurrent stone formers	Rarely — left to follow-up clinic	4	50.0
	Sometimes, if I remember	2	25.0
	Yes, always	1	12.5
	Not sure of the pathway	1	12.5

**Table 5:** Clinician-reported barriers to delivering secondary prevention advice. The item permitted multiple responses, so percentages sum to more than 100%. The abbreviation n denotes the number of respondents.

Barrier reported	n	%
Unclear whose responsibility it is	7	87.5
Other clinical priorities take precedence	5	62.5
No standard leaflet or resource available	4	50.0
Unsure of what advice to give	4	50.0
Not enough time during the encounter	3	37.5
Assumed it would be given by someone else	2	25.0
No time to document it separately	2	25.0
Patient in too much pain or distress to engage	0	0.0
Does not experience significant barriers	0	0.0

**Table 6:** Distribution of clinician self-rated confidence in delivering prevention advice. Confidence was rated on a five-point scale, where 1 indicated not confident and 5 indicated very confident. The abbreviation n denotes the number of respondents.

Self-rated confidence (1–5)	N	%
1 — not confident	1	12.5
2	6	75.0
3	1	12.5
4	0	0.0
5 — very confident	0	0.0
Mean score	2.0	—

**Table 7:** Free-text responses to the question asking what single change would most improve delivery of prevention advice. Five of eight respondents answered this item, and all five responses are reproduced verbatim. No responses were submitted to the second free-text item. Four of the five requested a printed patient information resource; the fifth requested both a resource and clarification of professional responsibility.

Respondent	Free-text response
1	Western Trust stone prevention booklets printed
2	If leaflets were readily available to be distributed
3	Automated patient letter
4	Leaflets readily available on the ward
5	Standard advice leaflet and instructions regarding who is responsible for giving this advice

 Appendix 3. Data collection proforma

**Table 8:** Variables recorded on the data collection proforma. The identical instrument was used in both audit cycles to permit direct comparison.

Domain	Variables recorded
Demographics	Age; sex
Presentation	Presentation setting (emergency department, elective, urology clinic); admission status
Stone characteristics	Stone location; maximum diameter (mm); presence of hydronephrosis
Investigation	Imaging modality performed; timing relative to presentation
Management	Analgesia administered; medical expulsive therapy given and agent used; definitive surgical management
Prevention (primary outcome)	Documented hydration advice; documented dietary advice; overall documented prevention advice; metabolic evaluation arranged
Follow-up	Follow-up arranged; modality of follow-up
History	Previous history of stone disease; prior prevention advice documented
Free text	Setting and manner in which prevention advice was delivered, where recorded
